# Diversity and selective sweep in the *OsAMT1;1 *genomic region of rice

**DOI:** 10.1186/1471-2148-11-61

**Published:** 2011-03-08

**Authors:** Zehong Ding, Chongrong Wang, Sheng Chen, Sibin Yu

**Affiliations:** 1 National Key Laboratory of Crop Genetic Improvement, and the College of Plant Science and Technology, Huazhong Agricultural University, Wuhan 430070, PR China; 2 School of Plant Biology, and International Centre for Plant Breeding Education and Research, The University of Western Australia, Crawley, WA 6009, Australia

## Abstract

**Background:**

Ammonium is one of the major forms in which nitrogen is available for plant growth. *OsAMT1;1 *is a high-affinity ammonium transporter in rice (*Oryza sativa *L.), responsible for ammonium uptake at low nitrogen concentration. The expression pattern of the gene has been reported. However, variations in its nucleotides and the evolutionary pathway of its descent from wild progenitors are yet to be elucidated. In this study, nucleotide diversity of the gene *OsAMT1;1 *and the diversity pattern of seven gene fragments spanning a genomic region approximately 150 kb long surrounding the gene were surveyed by sequencing a panel of 216 rice accessions including both cultivated rice and wild relatives.

**Results:**

Nucleotide polymorphism (Pi) of *OsAMT1;1 *was as low as 0.00004 in cultivated rice (*Oryza sativa*), only 2.3% of that in the common wild rice (*O. rufipogon*). A single dominant haplotype was fixed at the locus in *O. sativa*. The test values for neutrality were significantly negative in the entire region stretching 5' upstream and 3' downstream of the gene in all accessions. The value of linkage disequilibrium remained high across a 100 kb genomic region around *OsAMT1;1 *in *O. sativa*, but fell rapidly in *O. rufipogon *on either side of the promoter of *OsAMT1;1*, demonstrating a strong natural selection within or nearby the ammonium transporter.

**Conclusions:**

The severe reduction in nucleotide variation at *OsAMT1;1 *in rice was caused by a selective sweep around *OsAMT1;1*, which may reflect the nitrogen uptake system under strong selection by the paddy soil during the domestication of rice. Purifying selection also occurred before the wild rice diverged into its two subspecies, namely *indica *and *japonica*. These findings would provide useful insights into the processes of evolution and domestication of nitrogen uptake genes in rice.

## Background

Rice, one of the world's most important crops, is the staple food of over half of the world's population. Cultivated rice occurs in two forms, *Oryza sativa *and *Oryza glaberrima. Oryza sativa*, referred to as the Asian rice, is cultivated worldwide but distributed mainly in Asia and has two subspecies, *indica *and *japonica*, estimated to have diverged at least about 10,000 years ago [1,2]. The two subspecies are further divided into major subpopulations: *indica *into *aus *and *indica*, *japonica *into *temperate japonica*, *tropical japonica*, and the *aromatic *group [3]. It has been proposed that these subpopulations were independently domesticated from divergent pools of *O. rufipogon *[3,4].

During the process of domestication, genetic diversity in rice has reduced dramatically as a result of natural and artificial selection [5-7]. With the rapid development of molecular genomics, the path of domestication has been traced in detail through characterization of several domestication genes associated with artificial selection. For example, a major gene for grain shattering in rice, *shattering 4 *(*sh4*), has been characterized: nucleotide diversity of a ~50 kb region surrounding *sh4*, is nearly one-tenth of the average value across the entire chromosome 4 [8,9]. The non-shattering *sh4 *allele was fixed in all the *O. sativa *varieties surveyed. A selective sweep nearby the *sh4 *genomic region was probably due to strong artificial selection of the non-shattering allele, which confers a trait important to efficient grain harvest [10]. Similar findings have been reported in two other selection-related genes in rice, namely *Red pericarp *(*Rc*), which is responsible for pericarp colour, and *waxy *(*wx*), for glutinous grains. The two were under strong artificial selection given the human preference for white rice and low-amylose grains. As a result, the level of polymorphism in and around these two genes is markedly reduced [11,12]. Nucleotide diversity in the gene *teosinte branched 1 *(*tb1*), which is involved in plant architecture, is much lower in maize than in *teosinte*, the wild relative of maize. Wang et al. [13] showed that maize has retained only 3% of the diversity found in *teosinte *in the 5' non-transcribed region of *tb1*, again as a result of human selection. This selection effect led to a selective sweep of a 60-90 kb long region across the 5' promoter to the *tb1*-transcribed sequence [14,15]. Similarly, nucleotide variation in the gene *phytoene synthase *(*Y1*), which makes maize kernels yellow, was only 5.3% of that in the gene that makes them white, owing to the human preference for yellow grains, which have higher nutritional value [16]. Because of the effect of selection hitchhiking, nucleotide diversity was lower around the region of *Y1 *from ~600 kb downstream to ~200 kb upstream [17]. These results provide significant insights into the selective sweeps brought about by artificial selection during the process of crop domestication.

Ammonium is one of the major forms in which nitrogen is available for plant growth and reproduction. Ammonium absorption from soil is mediated by ammonium transporters (AMT) through both high-affinity and low-affinity transport systems. The high-affinity transport system plays a major role when ammonium in soil is at micromolar ammonium concentrations, and the low-affinity transport system mainly at millimolar or higher concentrations [18,19]. Phylogenetic analysis shows that the two systems are represented by AMT subfamilies AMT1 and AMT2 respectively [20,21]. Kaiser et al. [22] reported that ammonium uptake decreased by about 30% in a T-DNA insertion line of *AtAMT1;1 *compared to the wild-type, which encodes a high-affinity ammonium transporter in *Arabidopsis thaliana*. Other studies have also confirmed that the role of AMT1 in other species [23-26].

Rice carries at least twelve AMT genes [21], three of which, *OsAMT1;1 *to *OsAMT1;3*, have been identified as members of AMT1, each showing a distinct expression pattern: *OsAMT1;1 *shows constitutive expression in both shoots and roots; *OsAMT1;2 *shows root-specific and ammonium-inducible expression; and *OsAMT1;3 *shows root-specific and nitrogen-suppressible expression [27,28]. *OsAMT1;1 *has only one open reading frame encoding 499 amino acid residues. Its transcript was abundant in roots of plants that were grown under low levels of NH_4_^+ ^but decreased rapidly when the plants were transferred to a medium with high NH_4_^+ ^concentration [27]. Moreover, *OsAMT1;1 *expression was promoted by low ammonium in rice roots [28] and regulated by endogenous glutamine rather than by endogenous ammonium [29]. However, nucleotide variations in *OsAMT1;1 *and in other key genes in the nitrogen uptake and assimilation pathway are yet to be elucidated, and their evolutionary path from wild rice to cultivated rice remains unclear.

Improving the uptake and assimilation efficiency of nitrogen has been a major breeding target in rice, and success depends largely on discovering allelic variations of nitrogen metabolism genes in rice germplasm and understanding their adaptive significance. The objectives of the present study were 1) to investigate allelic variations of *OsAMT1;1*, 2) to analyse its pattern of polymorphism and its evolution, and 3) to define the extent of selective sweep around the *OsAMT1;1 *region in cultivated rice.

## Methods

### Rice materials

The nucleotide variation of the *OsAMT1;1 *gene was assessed in a panel of 216 *Oryza *accessions comprising 190 accessions of *O. sativa *(102 *indica*, 85 *japonica*, 2 aromatic, and 1 *aus *varieties), and 25 of wild rice (19 *O. rufipogon*, 5 *O. nivara*, and 1 *O. barthii*), and 1 of the African cultivated rice *O. glaberrima *(Additional file 1, Table S1). Most of the *O. sativa *accessions - 103 of them were of landraces (http://icgr.caas.net.cn/, Additional file 1, Table S1) - came from a collection of Chinese rice varieties estimated to represent about 70% of the total diversity in Chinese rice germplasm [30]. The other accessions of *O. sativa*, including 8 landraces, were selected from the OryzaSNP project http://www.oryzasnp.org/. The 25 wild relatives with different original sources were obtained from the International Rice Research Institute (IRRI).

Nucleotide diversity across the genomic region around *OsAMT1;1 *was also surveyed by sequencing seven gene segments from 94 accessions of *O. sativa *and 19 accessions of *O. rufipogon *(Additional file 1, Table S1).

### PCR and DNA sequencing

Nucleotide diversity in the gene *OsAMT1;1 *and the surrounding genomic region was assessed by PCR amplification and sequencing. Plant DNA was extracted from young leaves of each accession using cetyltrimethylammonium bromide (CTAB) method described by Rogers and Bendich [31]. All primers for the PCR were designed by Primer Premier 5.0 based on the Nipponbare genomic sequence available at the TIGR website http://rice.plantbiology.msu.edu/cgi-bin/gbrowse/rice/. For *OsAMT1;1*, four pairs of primers were used for PCR amplification to cover the entire coding sequence, ~1080 bp of the 5' promoter and ~110 bp of the 3' untranslated region. For each locus around *OsAMT1;1*, one pair of primer was designed to amplify fragments of the genomic DNA approximately 860-1060 bp long (Additional file 2, Table S2). To ensure that only the targeted genomic region is amplified, all PCR primers were confirmed by BLAST against the Nipponbare genomic sequence in the NCBI database http://blast.ncbi.nlm.nih.gov/Blast.cgi.

The PCR experiments were performed on 20 μL amplification reaction consisting of 0.75 unit of *Taq *polymerase (Takara, Dalian, China), 10 mM Tris-HCl, 50 mM KCl, 1.5 mM MgCl_2_, 0.2 mM dNTPs, 0.15 μM of each primer, and 50 ng of genomic DNA. PCR reactions were carried out in GeneAmp PCR System 9700 (Applied Biosystems, USA) under the following conditions: 5 min at 94°C, followed by 35 cycles of 1 min at 94°C, 1 min at a primer-specific temperature (Additional file 2, Table S2), and 1 min at 72°C and, for a final extension, 5 min at 72°C.

PCR products were directly sequenced with the BigDye Terminator Cycle Sequencing v3.1 (Applied Biosystems) after digestion and purification according to the manufacturer's specifications. Briefly, the PCR products were digested with Exonuclease I (Biolabs Inc.) and Shrimp Alkaline Phosphatase (Takara), and purified with 95% ethanol and 3 M sodium acetate (pH 5.2). Sequencing reactions were run on an automated capillary sequencer ABI 3730, and sequence contigs were assembled and edited using Sequencher 4.1.2 (Gene Codes Corporation, USA). To ensure reliability of the sequencing data, PCR amplicons were sequenced in both orientations, and any remaining ambiguities in the sequences resolved by repeated PCR amplification and re-sequencing. Almost all fragments were homozygous except two fragments from the two *O. rufipogon *accessions (DWR35 and DWR38) with heterozygous sites. PCR products of these two fragments were cloned into pGEM-T easy vectors (Promega, USA) and five independent clones were sequenced for each fragment. If two different alleles were found, the predominant allele of the five clones was arbitrarily chosen for further analysis [11].

### Data analysis

Sequences alignment was conducted with ClustalX 1.83 ftp://ftp-igbmc.u-strasbg.fr/pub/ClustalX/. The number of segregating sites and levels of nucleotide diversity Pi (π), the average number of nucleotide differences per site between two sequences [32], and θ, an estimate of 4Neμ, where Ne is the effective population size and μ is the mutation rate per nucleotide [33], were computed in DnaSP (version 5.10.00). Tajima's D test and Fu and Li's D* test were also performed in DnaSP for testing selections deviating from neutrality [34]. Haplotype analysis was conducted using TASSEL (version 2.0.1) [35]. To visualize the impact of sweep selection across the *OsAMT1;1 *genomic region, extended haplotype homozygosity (EHH) analysis was performed to measure the degree of linkage disequilibrium (LD), as sweep selection is expected to elevate the LD nearby the selected site. EHH was reported on a scale from 0 to 1, with 0 indicating that all extended haplotypes are different and 1 indicating that they are the same [36].

To investigate selection pressure on *OsAMT1;1*, *Ka*/*Ks *ratios (non-synonymous substitution rate / synonymous substitution rate) between *OsAMT1;1 *in rice and its homologs in the relative species were calculated using the method described by Goldman and Yang [37]. A *Ka*/*Ks *ratio significantly greater than one implies positive selection, less than one implies purifying selection, and a ratio of one may indicate neutrality [38].

## Results

### Sequence diversity at *OsAMT1;1*

**Figure 1:**
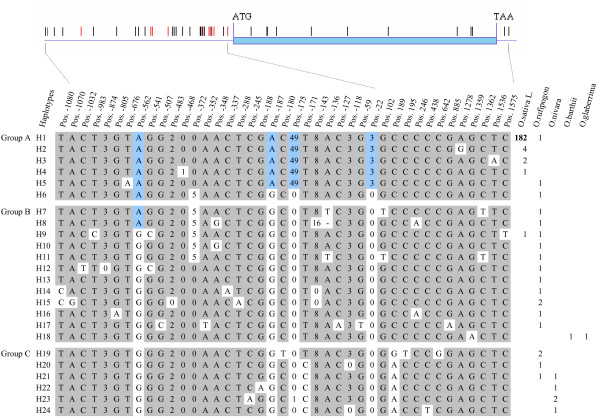
**Schematic position of nucleotide polymorphisms at *OsAMT1;1 *and haplotypes across the gene**. The short red and black lines above the model of the gene represent Indels and SNPs respectively, and the numbers below the model indicate their positions relative to the first base of the start codon (ATG) of *OsAMT1;1*. For example, pos. 874 means the distance between that SNP and the ATG of *OsAMT1;1 *is 874 bp in 'Nipponbare'. In each haplotype, letters indicate alternative nucleotides at a given position, numbers represent the size (bp) of a deletion (0 = no deletion), and the line of short dash in haplotype H8 represents a 1 bp deletion. Numbers to the right are those of the accessions contained in each haplotype.

Nucleotide polymorphism was examined in 216 accessions across the entire 2.68 kb length of *OsAMT1;1 *including a 1083 bp fragment of the 5' promoter region and a 111 bp fragment of the 3' untranslated region. The examination yielded 32 single nucleotide polymorphisms (SNP) and 9 insertions or deletions (Indels) in this 2.68-kb region (Figure 1), of which 30 polymorphism sites (25 SNPs and 5 Indels) were observed in the wild rice accessions and most were located in the non-coding region. Five polymorphism sites, at positions -562, -372, -187, -175, and -22 of the promoter region, occurred in high or intermediate frequencies in all the accessions (Figure 1), whereas at the coding region, ten SNPs - all synonymous variations - occurred rare frequency (well below 5%), indicating that the gene function had been conserved.

*Oryza rufipogon *was found to be at least 30 times as diverse as *O. sativa *across the entire sequenced region, including the coding and non-coding regions of *OsAMT1;1 *(Table 1). Nucleotide diversity π of the entire gene sequence was 0.00172, and θ was 0.00278 for *O. rufipogon*, while estimates of π for *indica *and *japonica *were much lower (π = 0.00006 and 0.00002 and θ = 0.00043 and 0.00007, respectively). These results indicate that the diversity of *OsAMT1;1 *was much less in *O. sativa *than in its wild relatives. In addition, nucleotide polymorphism in the coding region was much lower than that in the non-coding region for all accessions (Table 1). Nucleotide diversity in the coding region was about a third of that in the non-coding region in *O. rufipogon *and about half in cultivated rice (Table 1).

**Table 1:** Nucleotide diversity and neutral test at *OsAMT1;1*

Test set ^a^	Entire sequenced region (2688 bp)					Coding region (1494 bp)					Non-coding region (1194 bp)				
			
	S	π×10^3^	θ×10^3^	D1	D2	S	π×10^3^	θ×10^3^	D1	D2	S	π×10^3^	θ×10^3^	D1	D2
All (n = 216)	32	0.37	2.01	-2.33**	-2.01	10	0.16	1.12	-2.04*	-0.10	22	0.63	3.13	-2.15**	-2.57*
*O. rufipogon *(n = 19)	26	1.72	2.78	-1.59	-0.57	7	0.99	1.34	-1.22	0.79	19	2.65	4.60	-1.62	-1.16
*O. sativa *(n = 190)	7	0.04	0.45	-1.93*	-3.58**	1	0.03	0.11	-0.77	0.45	6	0.06	0.87	-1.89*	-4.08**
*Indica *(n = 102)	6	0.06	0.43	-1.90*	-3.67**	1	0.05	0.13	-0.69	0.49	5	0.08	0.81	-1.90*	-4.29**
*Japonica *(n = 85)	1	0.02	0.07	-0.90	0.50	0	0	0	NA	NA	1	0.04	0.17	-0.91	0.50

^a ^n in parentheses is the number of accessions used; S, number of segregating sites; π, nucleotide diversity per site; θ, Watterson's estimator; D1, Tajama's D; D2, Fu and Li's D*; NA, not applied; *, ** significant at 0.05 and 0.01(or 0.02) level for Tajama's D (or Fu and Li's D*), respectively.

Such extreme reduction in diversity of *OsAMT1;1 *was observed in both landraces of *O. sativa *and its elite varieties (Additional file 5, Figure S1). On the other hand, in the case of two other nitrogen-related genes (Loc_Os02 g50240 and Loc_Os01 g48960), the reduction in diversity in landraces and elite varieties was more modest; in fact in the case of Loc_Os01 g48960, the extent of diversity in landraces was nearly the same as that in elite lines of *O. sativa *(Additional file 5, Figure S1).

**Figure 2:**
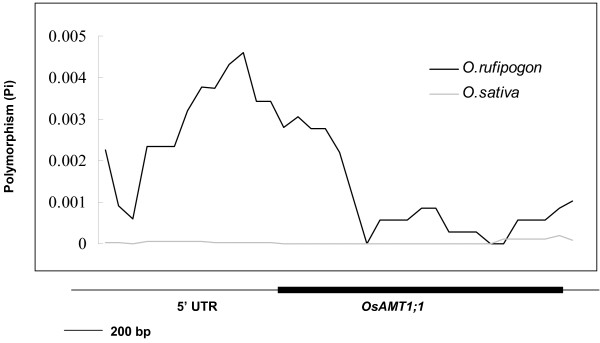
**Sliding-window analysis of nucleotide polymorphism (Pi)**. For the sliding-window analysis in *O. sativa *(190 accessions) and *O. rufipogon *(19 accessions), Pi was calculated for segments of 350 bp at 70 bp intervals. The black bar shows the exon of *OsAMT1;1*.

Results of Tajima's D and Fu and Li's D* tests for the entire region and the non-coding region of *OsAMT1;1 *were negative and significantly deviated from neutrality in all accessions, as well as in *O. sativa *samples. Fu and Li's D* values were not significant in the coding region in any of the accessions and subsamples (Table 1). Sliding window analysis of the pattern of polymorphism across the gene in *O. sativa *(190 accessions) and *O. rufipogon *(19 accessions) revealed a sharp peak at a point of about 200 bp upstream of the start codon of the gene (Figure 2). These results suggest a strong selection at *OsAMT1;1*, particularly in the 5' promoter region.

Notably, nine SNPs in the coding region of *OsAMT1;1 *that occurred in the wild relatives were all synonymous (Figure 1), suggesting that *OsAMT1;1 *is under purifying selection in the wild. To further validate this suggestion, *Ka*/*Ks *ratios were also calculated between *OsAMT1;1 *and its homologs in *O. barthii *(DWR50), *Sorghum bicolor *(Sb06 g022230), and *Zea mays *(GRMZM2G175140) from the TIGR website. The ratios ranged from 0.0010 to 0.0344, significantly less than 1.0 (Additional file 3, Table S3), suggesting strong purifying selection of *OsAMT1;1*.

### Haplotype variations across *OsAMT1;1*

A total of 24 haplotypes were identified in the entire region of gene *OsAMT1;1 *in the 216 accessions. Phylogenetic analysis showed that these distinct haplotypes fell into three groups (A, B and C). In group A, four haplotypes, H1 to H4, were detected in *O. sativa*. Most (182/190) accessions of the Asian cultivated rice (both *indica *and *japonica*) belonged to haplotype H1. Each of the haplotypes H2 to H5 in group A differed from H1 in only a single nucleotide site, whereas haplotype H6 differed from H1 in several sites (Figure 1). The 19 accessions of *O. rufipogon *fell into as many as 17 distinct haplotypes, indicating a high level of genetic variation in *O. rufipogon *(Figure 1). In group B, *O. glaberrima *and *O. barthii *had the same haplotype, H18, indicating their close genetic relationship. Five *O. nivara *and four *O. rufipogon *belonged to group C. As can be seen in Figure 1, almost all accessions of *O. sativa *are of haplotype H1, which incorporates the alleles at positions -562, -187, -175 and -22 in the 5' promoter region, whereas practically every accession of the wild rice is of a different haplotype. Incidentally, the one exception from *O. sativa *was of haplotype H9.

### Selective sweep surrounding *OsAMT1;1*

**Figure 3:**
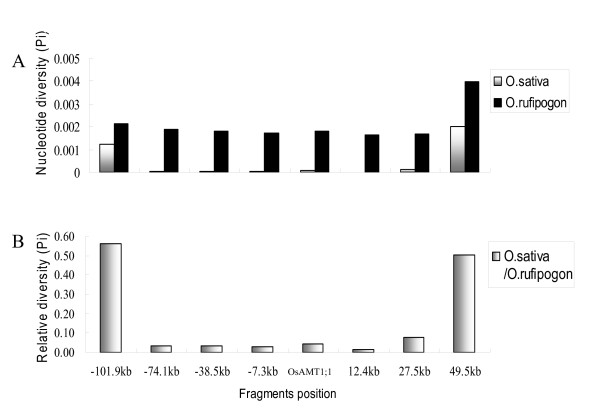
**Nucleotide diversity survey at *OsAMT1;1 *and at seven loci around it**. For each locus, nucleotide diversity was investigated by sequencing 94 accessions of *O. sativa *and 19 accessions of *O. rufipogon*. Positions (kb) of sequenced regions relative to *OsAMT1;1*. (A) Levels of nucleotide diversity (Pi) for *O. sativa *and *O. rufipogon*. (B) Ratio of nucleotide diversity at these regions between *O. sativa *and *O. rufipogon*.

To assess the impact of selection at *OsAMT1;1*, the extent of nucleotide polymorphism was examined at seven loci within a region around *OsAMT1;1 *approximately 150 kb long. The average nucleotide diversity (π) for *O. sativa *was low at five loci from -74.1 kb to 27.5 kb (Figure 3A and Table 2) but markedly high at the other two loci of -101.9 kb and 49.5 kb. The relative levels of nucleotide diversity between *O. sativa *and *O. rufipogon *were also calculated across the *OsAMT1;1 *genomic region. A striking reduction in the relative diversity (the ratio ranged from 0.012 to 0.077) was observed along the same region mentioned above (-74.1 kb to 27.5 kb) (Figure 3B and Table 2). These results indicate a selective sweep around *OsAMT1;1 *extending about 100 kb in length.

**Table 2:** Nucleotide diversity at *OsAMT1;1 *and at seven loci around it in *O. sativa *and *O. rufipogon*

Genes predicted	Position ^a^	Length ^b^	*O. sativa *(n = 94)^c^				*O. rufipogon *(n = 19)				
					
			Pi×10^3^	Hd	D1	D2	Pi×10^3^	Hd	D1	D2	The ratio of Pi ^d^
Glutaredoxin subgroup I	-101.9	853	1.21	0.516	1.38	-0.57	2.15	0.830	-2.02*	-3.11**	0.563
C3HC4 type zinc finger family	-74.1	757	0.06	0.042	-1.39	-2.80*	1.87	0.766	-1.31	-1.44	0.032
Lipase class 3 family	-38.5	756	0.06	0.042	-1.39	-2.80*	1.81	0.754	-1.63	-2.17	0.033
Cupin superfamily	-7.3	863	0.05	0.021	-1.39	-2.80*	1.72	0.865	-1.20	-1.44	0.029
*OsAMT1;1*	0	2688	0.08	0.123	-2.00*	-3.13*	1.72	0.982	-1.59	-0.57	0.047
Hypothetical protein	12.4	877	0.02	0.021	-1.03	-2.02	1.65	0.807	-1.25	-0.88	0.012
Transcriptional corepressor LEUNIG	27.5	638	0.13	0.042	-1.79*	-3.84**	1.69	0.678	-1.21	-1.51	0.077
Expressed protein	49.5	832	2.01	0.576	0.52	-1.40	3.99	0.906	-0.50	-0.41	0.504

^a ^Position of sampled loci as far to the first nucleotide of *OsAMT1;1 *that was considered as 0 kb; ^b ^Length (bp) of sampled alignment regions is relative to Nipponbare genomic sequence; ^c ^Pi (π), nucleotide diversity per site; Hd, haplotype diversity; D1, Tajama's D; D2, Fu and Li's D*; *, ** significant at 0.05 and 0.02 level, respectively; ^d ^The relative ratio of Pi for *O. sativa *to *O. rufipogon*.

Similar patterns were drawn from Hd, a measure of haplotype diversity, the value of which for *O. sativa *was markedly lower for the five loci along the stretch from -74.1 kb to 27.5 kb than that for either of the loci at -101.9 kb or 49.5 kb. However, Hd for *O. rufipogon *was much higher across all six loci including the gene *OsAMT1;1 *(Table 2). The values of Fu and Li's D* tests were significantly negative for the five loci (-74.1 kb to 27.5 kb) in *O. sativa*, which is a signature of selection in *OsAMT1;1 *genomic region from *O. rufipogon *to *O. sativa *(Table 2).

**Figure 4:**
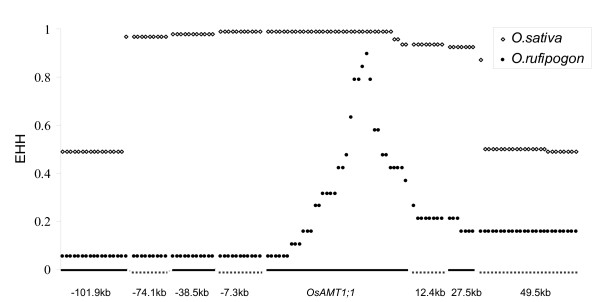
**Extended haplotype homozygosity (EHH) analysis across the *OsAMT1;1 *genomic region**. White diamonds and black dots indicate EHH values for *O. sativa *and *O. rufipogon *respectively. Broken horizontal lines along the X-axis indicate the polymorphisms corresponding to specific sites relative to *OsATM1;1*.

Lastly, the extent of linkage disequilibrium (LD) was measured by calculating the extended haplotype homozygosity (EHH) for *O. sativa *and *O. rufipogon*. The average EHH value was 0.926 in *O. sativa *across the region from -74.1 kb to 27.5 kb but abruptly dipped to 0.489 at the other two loci (-101.9 kb and 49.5 kb). The high value of LD along a distance of ~100 kb (from -74.1 kb to 27.5 kb) in *O. sativa *and the marked reduction in LD in *O. rufipogon *on either side of the core location in the promoter of *OsAMT1;1 *(Figure 4) support the idea of a strong selection acting on *OsAMT1;1*.

## Discussion

### Diversity and selection of *OsAMT1;1*

Artificial selection is believed to have played an important role in the severe reduction of genetic diversity in cultivated rice during its domestication from its wild ancestors. *OsATM1;1 *plays a key role in ammonium uptake and assimilation in rice but whether *OsATM1;1 *and other nitrogen-uptake genes were subjected to selection pressure during the domestication process is an open question. In the present study, we sequenced the entire gene *OsAMT1;1 *in a panel of 216 rice accessions, drawn mostly from Chinese cultivated rice (*O. sativa*), and observed the predominance of one major haplotype (H1) and extreme reduction in genetic diversity of *OsAMT1;1 *in cultivated rice compared to that in its wild progenitor *O. rufipogon*. The level of nucleotide polymorphism (π = 0.00006) in *OsAMT1;1 *in *O. sativa *was also markedly lower than that in 111 randomly chosen gene fragments (π = 0.0023) [6] and in 10 reference genes from *O. sativa *(π = 0.0037) [7]. This severe reduction of genetic diversity in cultivated rice and *Ka*/*Ks *ratios far below 1.0 (Additional file 3, Table S3) are probably characteristic of very strong selection pressure acting on *OsATM1;1*.

The severe reduction of genetic diversity and predominance of a single common haplotype in both subspecies may be a consequence of strong selection for the promoter region that potentially affects transcription of *OsAMT1;1*. This strong selection for the non-coding regions of *OsATM1;1 *in *O. sativa *is supported by the significantly negative values of Tajima's D and Fu and Li's D* tests (Table 1). We found that *O. sativa *specifically carried three polymorphic sites differing from the most wild rice accessions at positions of -22, -175, and -187 that were adjacent to several core domains such as TATA box in the promoter, which is a *cis*-regulatory DNA region that facilitates transcription of a particular gene for functional adaptation. We observed different expression levels of *OsAMT1;1 *in several near-isogenic lines that carried the alleles of *OsAMT1;1 *from *O. rufipogon *and *O. sativa *in the similar genetic background (Additional file 6, Figure S2). The expression of the *O. rufipogon *alleles in shoot of the near-isogenic line was significantly higher than that of ZS97. However, further investigation is necessary to explain the advantages conferred by the selected allelic variations in *OsAMT1;1 *during domestication. Several studies have shown that sequence variations in the promoter region result in differential regulation of transcription. For example, Crawford et al. [39] revealed that the *lactate dehydrogenase-B *(*Ldh-B*) proximal promoter sequence affected transcriptional processes and constituted a phenotypic change that was subject to natural selection by temperature differences. Konishi et al. [40] demonstrated that a single-nucleotide polymorphism in the 5' regulatory region of the QTL for seed shattering in chromosome 1 (*qSH1*) causes loss of seed shattering and was therefore the target of artificial selection during rice domestication. Similarity, the promoter and proximal intergenic region of *tb1 *was the target of selection, and a selective sweep ~90 kb in length across this 5' region occurred during maize domestication [14,15].

Furthermore, the LD value was high along a ~100 kb region in *O. sativa*, but fell sharply in *O. rufipogon*. Fu and Li's D* tests were significant for five loci located in a region from -74.1 kb to 27.5 kb. These observations indicate a selective sweep within and nearby *OsAMT1;1 *(Figure 4 and Table 2). Many agronomically important genes such as *sh4 *and *waxy *were under strong artificial selection leading to a selective sweep around the site of those genes. We infer a similar selective sweep around *OsAMT1;1 *caused largely by natural selection for an ammonium transporter.

### Reasons for *OsAMT1;1 *selection

The common wild rice *O. rufipogon *is found in ditches, pools, and other sites with shallow, standing, or slow-running water. In these natural habitats, the level of nitrogen is extremely low [41]. Nitrogen, either as ammonia or as nitrite, is present only in traces both in water and in the sediment around such habitats. Therefore, a nitrogen uptake system that can manage to obtain nitrogen from such infertile soil was most likely a target of selection during the transition from the wild progenitor to cultivated rice. In addition, until the arrival of nitrogenous fertilizers, the major sources of nitrogen were animal excreta and plant and animal residues, the ultimately usable forms being ammonia, acid amides, and urea. As a result, ammonium was the main form of nitrogen in anaerobic rice paddy environments [42] - these habitats remained unchanged for thousands of years until the 20th century, which saw large-scale application of chemical fertilizers. Even for current rice cultivation in paddy fields, ammonium remains the major form of inorganic nitrogen, making rice almost dependent on ammonium nutrition during a large part of the cropping season. In terms of the efficiency of fertilizer use, ammonium is superior to nitrate in paddy soils [43]. Therefore, a high-affinity ammonia transporter such as *OsAMT1;1 *for ammonium uptake in rice [27,28], would have proved advantageous in adapting to domestication and remained under continual selection pressure in low-nitrogen soils.

We note that of 21 annotated genes located in the ~150 kb genomic region around *OsAMT1;1 *based on the TIGR website (Additional file 4, Table S4), nine are reported to be involved in plant responses to such biotic and/or abiotic stresses as extreme temperatures, drought, excess salt, and infectious pathogens [44-52]. Rice has adapted to changes in several environmental features (e.g. soil, water, photoperiod, and pathogens). Selection for these genes nearby *OsAMT1;1 *rather than *OsAMT1;1 *alone could not be excluded as the cause of the selective sweep around the *OsAMT1;1 *genomic region. However, inadequate soil nitrogen should be the most widespread selection pressure in operation for both subspecies, irrespective of their locations. We therefore suggest that fixation of the alleles of *OsAMT1;1 *common to the two subspecies *indica *and *japonica *is the result of a combined force of natural selection for more than one trait. Unlike the domestication genes, *rc*, *sh4*, and *wx *that were involved in traits strongly preferred by humans [9], the gene *OsAMT1;1 *may have been selected as a results of soils deficient in nitrogen.

### Evolutionary process of *OsAMT1;1*

In the present study, haplotype H1 is predominant and fixed at *OsAMT1,1 *in both subspecies (*indica *and *japonica*). This dominant haplotype was also found in *O. rufipogon *albeit in very low frequency - only one *O. rufipogon *accession, namely DWR25 from Malaysia, carried it - suggesting a possibility that haplotype H1 first occurred in one of the subspecies of *O. sativa *and introgressed into the wild rice. We also observed that one *O. rufipogon *accession (DWR27 from China) had only one SNP (T/A) at position -676 compared to H1 (Figure 1). This instance of high diversity of the gene in the wild relatives but uniform outcome because all mutations were synonymous suggests another possibility, namely that the haplotype H1 of *OsAMT1;1 *occurred first in one of the accessions of *O. rufipogon *(e.g. DWR27), and underwent strong selection pressure during domestication. A similar pattern was reported in the non-shattering allele (*sh4*) fixed in 208 rice cultivars [53]. Actually, two models, snowballing model and combination model, were previously proposed considering single and multiple origins of cultivated rice, respectively [9]. These two models reconciled the data for *sh4*. These models could also be applied in *OsAMT1;1 *as *OsAMT1;1 *and *sh4 *shared the same features in terms of the nucleotide diversity. However, current sequence data from *OsAMT1;1 *and *sh4 *might be not strong enough to distinctly support which model is correct. With the rapid development of sequencing technology, more genes similar to *OsAMT1;1 *and *sh4 *would be discovered in the rice genome. Further cloning and functional characterization of these genes will help test the models of rice domestication.

Additionally, although genetic diversity in *indica *was double or triple of that in *japonica*, genetic diversity in common wild rice *O. rufipogon *around *OsAMT1;1 *was more than 30 times higher than that both *indica *and *japonica *rice (Table 1). It is remarkable that genetic diversity in two other nitrogen-related genes decreased gradually from the wild relatives to the cultivated rice, whereas the reduction was extreme in the case of *OsAMT1;1 *(Additional file 5, Figure S1). Therefore the selection around *OsAMT1;1 *may have occurred before the divergence of *indica *and *japonica*. Several domestication genes such as *rc *and *waxy *found in domesticated forms with their favorable alleles occurred first in the *japonica *cultivars and experienced the selective sweep under strong artificial selection during domestication, causing the alleles to become fixed in all subpopulations of rice [12,54]. *OsAMT1;1 *with only one predominant haplotype in both *indica *and *japonica *cultivars even in landraces supports the contention that the gene was first under purifying selection in the wild and then under positive selection during domestication (Additional file 3, Table S3, and Figure 4). However, further investigations using many more samples drawn from locations across the world are required for more definitive answers.

## Conclusions

The ammonium transporter *OsAMT1;1 *showed abundant genetic diversity in *O. rufipogon *-- diversity that has been dramatically lost in *O. sativa *owing to natural and artificial selection during the domestication of rice. This severe reduction in the extent of variation in nucleotides of *OsAMT1;1 *in the cultivated rice (*O. sativa*) extends to a genomic region about 100 kb long, indicating a selective sweep around *OsAMT1;1*. This study demonstrates that the selective sweep was caused by strong selection within or nearby the ammonium transporter during the process of domestication of rice from its wild progenitors to cultivated rice. Understanding the diversity pattern of the ammonium transporter gene in rice not only provides valuable information on the allelic distribution of the nitrogen-uptake gene, but also gives important insights into the process of domestication of cultivated rice. For example, given the alleles fixed at *OsAMT1;1 *in *O. sativa*, it is possible to discover novel alleles in wild relatives to broaden the genetic variation for improving the efficiency of nitrogen uptake in plants. It will also be worthwhile to elucidate the pattern of diversity of homologs of AMT1 in other crops or of other nitrogen genes in rice.

## Authors' contributions

ZD conducted DNA sequencing, analyzed data and drafted the manuscript; CW conducted accessions collection; SC participated in development of the manuscript; SY designed the study, analyzed data, and wrote the paper; all authors read and approval the manuscript.

## Supplementary Material

Additional file 1 **Table S1: Details of sources, classification, and haplotypes of accessions of cultivated rice and wild rice used in this study**. Click here for file

Additional file 2 **Table S2: Primers used for PCR amplification and sequencing**. The targeted genes with positions relative to *OsAMT1;1*, annealing temperature, and predicted product size for amplification are listed. Click here for file

Additional file 3 **Table S3: *Ka*/*Ks *test for *OsAMT1;1***. *Ka*, *Ks *represent non-synonymous and synonymous substitutions rate respectively. Click here for file

Additional file 4 Table S4: Summary of 21 annotated genes surrounding *OsAMT1;1*. Click here for file

Additional file 5 **Figure S1: Comparison of nucleotide diversity in *OsAMT1;1 *and two other genes related to nitrogen metabolism in *O. rufipogon*, landraces, and elite rice**. (n) = no. of the samples assayed in each subgroup. Click here for file

Additional file 6 **Figure S2: Expression of *OsAMT1;1 *in paired near-isogenic lines under low nitrogen**. NIL1 and NIL2 represent that near-isogenic line carried the *OsAMT1;1 *allele from 'ACC10' (*O. rufipogon*) and from 'Nipponbare' (*japonica*), respectively within the same genetic background of 'Zhenshan97' (ZS97). Young seedlings at the two-leaf stage were transferred to a Yoshida nutrient solution with one modification representing low nitrogen (0.15 mM (NH_4_)_2_SO_4_). The nutrient solution was replaced every three days. Seedlings were grown in the nutrient solution for seven days, after which their roots and shoots were harvested separately, frozen in liquid nitrogen, and stored at -70°C until required for RNA isolation. Total RNA was isolated using Trizol reagent (Invitrogen). qRT-PCR (quantitative real-time PCR) was performed using the forward primer 5'-CTGGGGTTGGTGGGTTCA-3' and reverse primer 5'-CACTTGGTTGTTGCTGTTGGAG-3' for *OsAMT1;1 *and the primers 5'-AACCAGCTGAGGCCCAAGA-3' and 5'-ACGATTGATTTAACCAGTCCATGA-3' for rice *ubiquitin *gene, which served as the internal control. Relative expression values are given as means ± standard error from three biological replications each with three technical repeats, and the *p *value next to each bar represents the results of *t *test between a given NIL and ZS97. Error bars indicate standard error. Click here for file
