# Supplementary material for: Diversity and selective sweep in the OsAMT1;1 genomic region of rice
Source: BMC Evol Biol. 2011 Mar 8;11:61. doi: 10.1186/1471-2148-11-61 (PMC3062601; doi:10.1186/1471-2148-11-61)
Supplement: Additional file 3 — Table S3: Ka/Ks test for OsAMT1;1. Ka, Ks represent non-synonymous and synonymous substitutions rate respectively. [file 1471-2148-11-61-S3.DOC]

Additional file 3 -Table S3 Summary of non-synonymous (*Ka*) and synonymous (*Ks*) substitutions rate in *OsAMT1;1*

| Source of homolog ^a^ | *Ka* | *Ks* | *Ka*/*Ks* | *P* value (Fisher) |
| --- | --- | --- | --- | --- |
| *O.barthii* | 1.13E-05 | 0.0113 | 0.0010 | 0.023 |
| *Sorghum bicolor* | 0.0367 | 1.0668 | 0.0344 | 1.98E-104 |
| *Zea mays* | 0.0350 | 1.1112 | 0.0315 | 2.05E-109 |

^a^ Homolog in *O. barthii* (DWR50 in this study), *Sorghum bicolor* (Sb06g022230) and *Zea mays* (GRMZM2G175140) from the TIGR website.
