# Supplementary material for: Diversity and selective sweep in the OsAMT1;1 genomic region of rice
Source: BMC Evol Biol. 2011 Mar 8;11:61. doi: 10.1186/1471-2148-11-61 (PMC3062601; doi:10.1186/1471-2148-11-61)
Supplement: Additional file 4 — Table S4: Summary of 21 annotated genes surrounding OsAMT1;1. [file 1471-2148-11-61-S4.DOC]

**Additional file 4-Table S4** **Summary of 21 annotated genes surrounding *OsAMT1;1*.**

| Locus ID | Position (kb) ^a^ | Annotation from TIGR | Putative function |
| --- | --- | --- | --- |
| LOC_Os04g42940 | -99.9 | expressed protein | unknown |
| LOC_Os04g42950 | -95.2 | MYB family transcription factor | response to salt stress and pathogen attack (Vailleau et al. 2002; Nagaoka and Takano 2003) |
| LOC_Os04g42960 | -83.1 | Lung seven transmembrane receptor domain containing protein | unknown |
| LOC_Os04g42970 | -79.9 | hypothetical protein | unknown |
| LOC_Os04g42980 | -74.1 | C3HC4 type zinc finger family protein | response to stress, hormone and light (Ma et al. 2009) |
| LOC_Os04g42990 | -64.9 | suppressor of stem-loop protein 1 | promote DNA repair and is essential for translation initiation (Yoon et al. 1992) |
| LOC_Os04g43010 | -48.5 | retrotransposon protein | unknown |
| LOC_Os04g43020 | -46.7 | protein kinase | response to salt, pathogen invasion, hormones, temperature stress and nutrient deprivation (Stone and Walker 1995; Guo et al. 2001) |
| LOC_Os04g43030 | -38.5 | lipase class 3 family protein | involve in leaf senescence, regulate empty-glume fate and spikelet development (Li et al. 2009) |
| LOC_Os04g43040 | -31.6 | expressed protein | unknown |
| LOC_Os04g43050 | -26.1 | Dicer | response to drought, cold, salt and disease resistance (Liu et al. 2007; Liu et al. 2009) |
| LOC_Os04g43060 | -7.3 | enzyme of the cupin superfamily protein | response to high temperature, salt and oxidative stress (Khuri et al. 2001; Dunwell et al. 2004) |
| LOC_Os04g43070 | 0 | ammonium transporter protein (*OsAMT1;1*) | response to nitrogen (Kumar et al. 2003; Sonoda et al. 2003) |
| LOC_Os04g43080 | 12.4 | hypothetical protein | unknown |
| LOC_Os04g43090 | 18.1 | expressed protein | unknown |
| LOC_Os04g43100 | 21.0 | conserved hypothetical protein | response to stress such as physical wounding and pathogen attack (Keith et al. 1991) |
| LOC_Os04g43110 | 23.3 | hypothetical protein | unknown |
| LOC_Os04g43120 | 24.7 | hypothetical protein | unknown |
| LOC_Os04g43130 | 27.5 | transcriptional corepressor LEUNIG | response to disease resistance, salt, wounding and drought (Gonzalez et al. 2007; Sitaraman et al. 2008) |
| LOC_Os04g43140 | 35.1 | DEAD-box ATP-dependent RNA helicase | response to low temperature, high salinity, pathogen infection and oxidative stress (Gong et al. 2005; Kant et al. 2007; Li et al. 2008) |
| LOC_Os04g43150 | 43.2 | nuclear transport factor 2 | involve in signal transduction such as pathogen signaling (Doczi et al. 2007; He et al. 2010) |

^a^ distance relative to *OsAMT1;1*.

**References cited:**

Doczi R, Brader G, Pettko-Szandtner A, Rajh I, Djamei A, Pitzschka A, Teige M and Hirt H. 2007. The Arabidopsis mitogen-activated protein kinase kinase MKK3 is upstream of group C mitogen-activated protein kinases and participates in pathogen signaling. *The Plant Cell* **19**: 3266–3279

Dunwell JM, Purvis A, Khuri S. 2004. Cupins: the most functionally diverse protein superfamily? *Phytochemistry* **65**: 7–17

Gong ZZ, Dong CH, Lee H, Zhu JH, Xiong LM, Gong DM, Stevenson B and Zhu JK. 2005. A dead box RNA helicase is essential for mRNA export and important for development and stress responses in Arabidopsis. *The Plant Cell* **17**: 256–267

Gonzalez D, Bowen AJ, Carroll TS and Conlan RS. 2007. The transcription corepressor LEUNIG interacts with the histone deacetylase HDA19 and mediator components MED14 (SWP) and CDK8 (HEN3) to repress transcription. *Molecular and Cellular Biology* **27(15)**: 5306–5315

Guo Y, Halfter U, Ishitani M and Zhu JK. 2001. Molecular characterization of functional domains in the protein kinase SOS2 that is required for plant salt tolerance. *The Plant Cell* **13**: 1383–1400

He HJ, Wang Q, Zheng WW, Wang JX, Song QS, Zhao XF. 2010. Function of nuclear transport factor 2 and Ran in the 20E signal transduction pathway in the cotton bollworm, *Helicoverpa armigera*. *BMC Cell Biology* **11**: 1

Kant P, Kant S, Gordon M, Shaked R and Barak S. 2007. STRESS RESPONSE SUPPRESSOR1 and STRESS RESPONSE SUPPRESSOR2, two DEAD-box RNA helicases that attenuate Arabidopsis responses to multiple abiotic stresses. *Plant Physiology* **145(3)**: 814–830

Keith B, Dong XN, Ausubel FM and Fink GR. 1991. Differential induction of 3-deoxy-D-arabino-heptulosonate 7-phosphate synthase genes in *Arabidopsis thaliana* by wounding and pathogenic attack. *Proc Nati Acad Sci USA* **88(19)**: 8821–8825

Khuri S, Bakker FT and Dunwell JM. 2001. Phylogeny, function, and evolution of the cupins, a structurally conserved, functionally diverse superfamily of proteins. *Molecular Biology and Evolution* **18(4)**: 593–605

Kumar A, Silim SN, Okamota M, Siddiqi MY and Glass ADM. 2003. Differential expression of three members of the AMT1 gene family encoding putative high-affinity NH_4_^+^ transporters in roots of *Oryza sativa* subspecies *indica*. *Plant, Cell and Environment* **26**: 907–914

Li DY, Liu HZ, Zhang HJ, Wang XE and Song FM. 2008. *OsBIRH1*, a DEAD-box RNA helicase with functions in modulating defense responses against pathogen infection and oxidative stress. *Journal of Experimental Botany* **59(8)**: 2133–2146

Li HG, Xue DW, Gao ZY, Yan MX, Xu WY, Xing Z, Huang DN, Qian Q and Xue YB. 2009. A putative lipase gene EXTRA GLUME1 regulates both empty-glume fate and spikelet development in rice. *The Plant Journal* **57**: 593–605

Liu B, Chen ZY, Song XW, Liu CY, Cui X, Zhao XF, Fang J, Xu WY, Zhang HY, Wang XJ, Chu CC, Deng XW, Xue YB and Cao XF. 2007. *Oryza sativa* Dicer-like4 reveals a key role for small interfering RNA silencing in plant development. *Plant Cell* **19(9)**: 2705–2718

Liu QP, Feng Y and Zhu ZJ. 2009. Dicer-like (DCL) proteins in plants. *Funct Integr Genomics* **9**: 277–286

Ma K, Xiao JH, Li XH, Zhang QF, Lian XM. 2009. Sequence and expression analysis of the C3HC4-type Ring finger gene family in rice. *Gene* **444**: 33–45

Nagaoka S and Takano T. 2003. Salt tolerance-related protein STO binds to a Myb transcription factor homologue and confers salt tolerance in Arabidopsis. *Journal of Experimental Botany* **54(391)**: 2231–2237

Sitaraman J, Bui M and Liu ZC. 2008. LEUNIG_HOMOLOG and LEUNIG perform partially redundant functions during Arabidopsis embryo and floral development. *Plant Physiology* **147**: 672–681

Sonoda Y, Ikeda A, Saiki S, Wiren N, Yamaya T and Yamaguchi J. 2003. Distinct expression and function of three ammonium transporter genes (*OsAMT1;1-1;3*) in rice. *Plant Cell Physiology* **44(7)**: 726–734

Stone JM and Walker JC. 1995. Plant protein kinase families and signal transduction. *Plant Physiolgy* **108**: 451–457

Vailleau F, Daniel X, Tronchet M, Montillet JL, Triantaphylides C and Roby D. 2002. A R2R3-MYB gene, *AtMYB30*, acts as a positive regulator of the hypersensitive cell death program in plant in response to pathogen attack. *Proc Nati Acad Sci USA* **99(15)**: 10179–10184

Yoon H, Miller SP, Pabich EK and Donahue TF. 1992. SSL1, a suppressor of a HIS4 5’-UTR stem-loop mutation, is essential for translation initiation and affects UV resistance in yeast. *Genes and Development* **6**: 2463–2477
