# Supplementary figures and images for: Diversity and selective sweep in the OsAMT1;1 genomic region of rice
Source: BMC Evol Biol. 2011 Mar 8;11:61. doi: 10.1186/1471-2148-11-61 (PMC3062601; doi:10.1186/1471-2148-11-61)

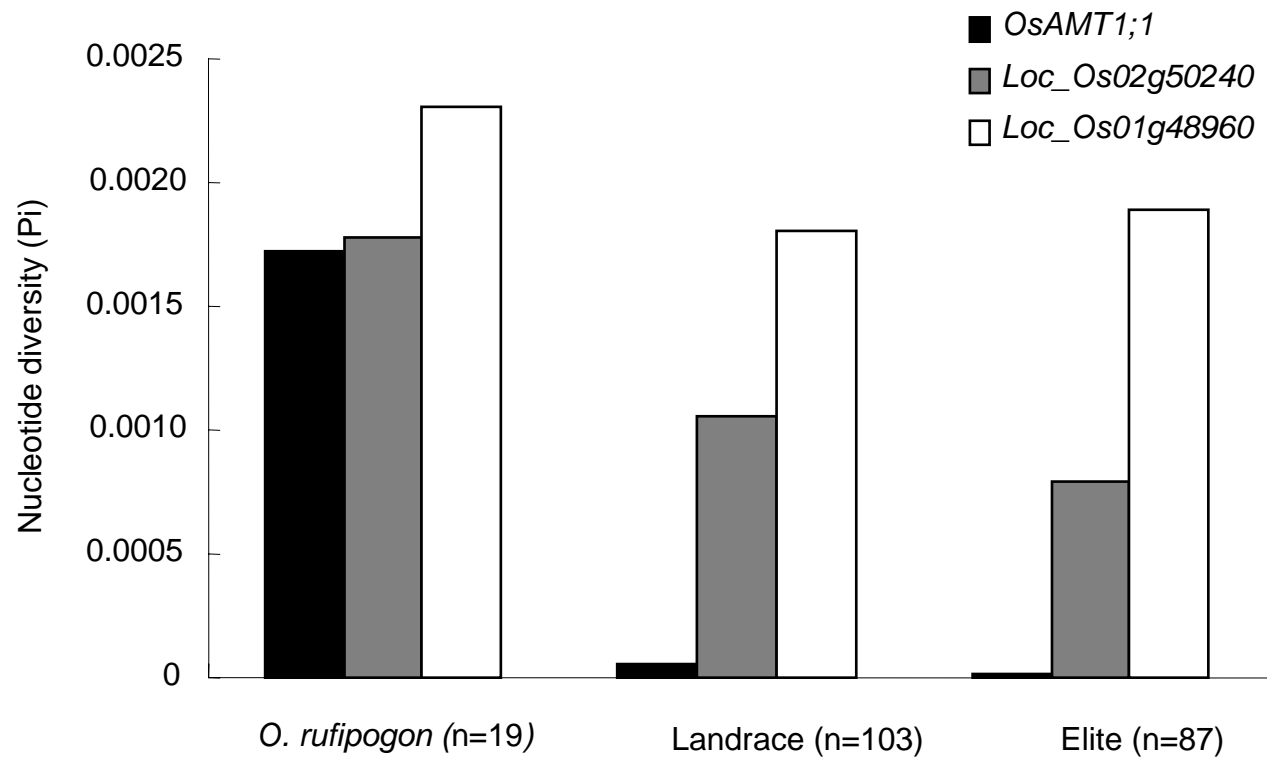

Supplement: Additional file 5 — Figure S1: Comparison of nucleotide diversity in OsAMT1;1 and two other genes related to nitrogen metabolism in O. rufipogon, landraces, and elite rice. (n) = no. of the samples assayed in each subgroup. [file 1471-2148-11-61-S5.PDF]

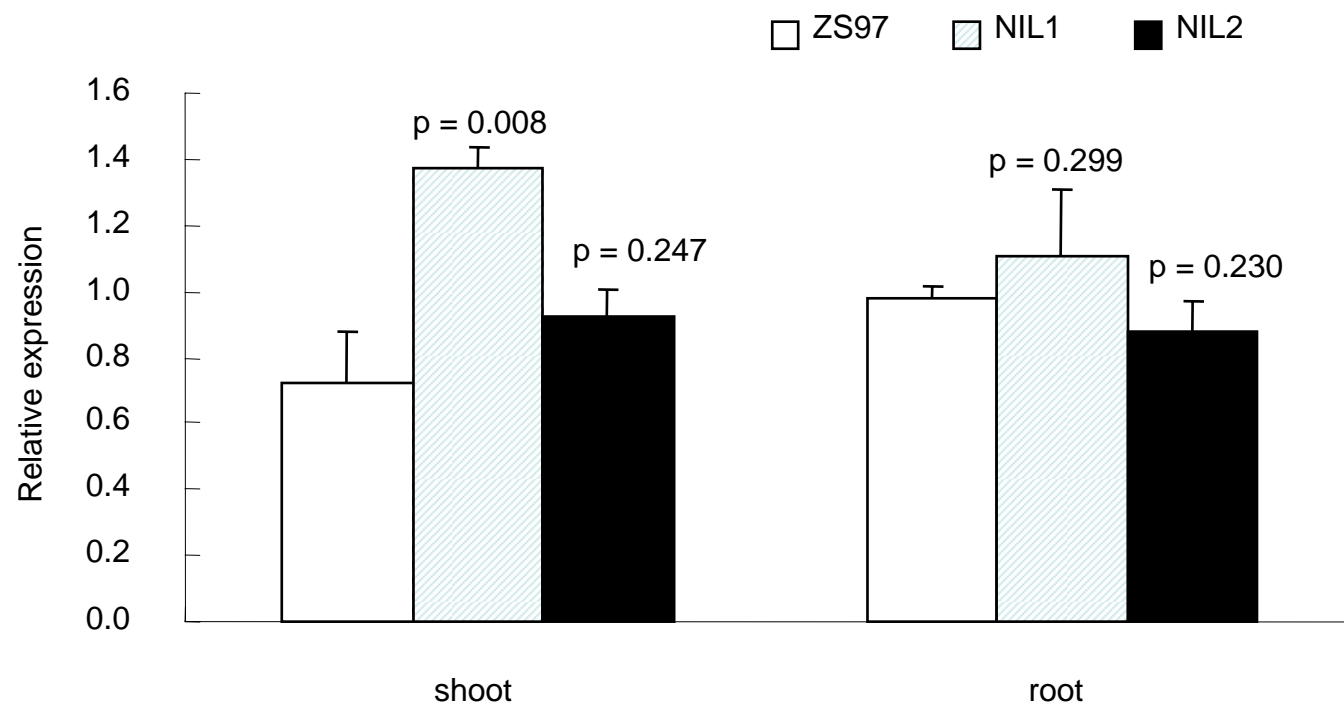

Supplement: Additional file 6 — Figure S2: Expression of OsAMT1;1 in paired near-isogenic lines under low nitrogen. NIL1 and NIL2 represent that near-isogenic line carried the OsAMT1;1 allele from 'ACC10' (O. rufipogon) and from 'Nipponbare' (japonica), respectively within the same genetic background of 'Zhenshan97' (ZS97). Young seedlings at the two-leaf stage were transferred to a Yoshida nutrient solution with one modification representing low nitrogen (0.15 mM (NH4)2SO4). The nutrient solution was replaced every three days. Seedlings were grown in the nutrient solution for seven days, after which their roots and shoots were harvested separately, frozen in liquid nitrogen, and stored at -70°C until required for RNA isolation. Total RNA was isolated using Trizol reagent (Invitrogen). qRT-PCR (quantitative real-time PCR) was performed using the forward primer 5'-CTGGGGTTGGTGGGTTCA-3' and reverse primer 5'-CACTTGGTTGTTGCTGTTGGAG-3' for OsAMT1;1 and the primers 5'-AACCAGCTGAGGCCCAAGA-3' and 5'-ACGATTGATTTAACCAGTCCATGA-3' for rice ubiquitin gene, which served as the internal control. Relative expression values are given as means ± standard error from three biological replications each with three technical repeats, and the p value next to each bar represents the results of t test between a given NIL and ZS97. Error bars indicate standard error. [file 1471-2148-11-61-S6.PDF]
